# Antioxidant Capacity of Free and Bound Phenolics from Olive Leaves: In Vitro and In Vivo Responses

**DOI:** 10.3390/antiox12122033

**Published:** 2023-11-23

**Authors:** Ting Li, Wenjun Wu, Jianming Zhang, Qinghang Wu, Shenlong Zhu, Erli Niu, Shengfeng Wang, Chengying Jiang, Daqun Liu, Chengcheng Zhang

**Affiliations:** 1 Food Science Institute, Zhejiang Academy of Agricultural Sciences, Hangzhou 310021, China; lt1345600496@126.com (T.L.); zhangjianming@zaas.ac.cn (J.Z.); hang9799@outlook.com (Q.W.); 2 College of Food and Health, Zhejiang A&F University, Hangzhou 311300, China; 3 Gansu Research Academy of Forestry Science and Technology, Lanzhou 730020, China; wuwenjun121@163.com (W.W.); jcytxb@126.com (C.J.); 4 Institute of Crop and Nuclear Technology Utilization, Zhejiang Academy of Agricultural Sciences, Hangzhou 310021, China; zhusl@zaas.ac.cn (S.Z.); niuerli@zaas.ac.cn (E.N.); 5 Research Center of Analysis and Measurement, Zhejiang University of Technology, Hangzhou 310014, China; wsf1027@zjut.edu.cn

**Keywords:** olive leaves, free phenolics, bound phenolics, antioxidant properties, oxidative stress

## Abstract

Olive leaves are rich in phenolic compounds. This study explored the chemical profiles and contents of free phenolics (FPs) and bound phenolics (BPs) in olive leaves, and further investigated and compared the antioxidant properties of FPs and BPs using chemical assays, cellular antioxidant evaluation systems, and in vivo mouse models. The results showed that FPs and BPs have different phenolic profiles; 24 free and 14 bound phenolics were identified in FPs and BPs, respectively. Higher levels of phenolic acid (i.e., sinapinic acid, 4-coumaric acid, ferulic acid, and caffeic acid) and hydroxytyrosol were detected in the BPs, while flavonoids, triterpenoid acids, and iridoids were more concentrated in the free form. FPs showed a significantly higher total flavonoid content (TFC), total phenolic content (TPC), and chemical antioxidant properties than those of BPs (*p* < 0.05). Within the range of doses (20–250 μg/mL), both FPs and BPs protected HepG2 cells from H_2_O_2_-induced oxidative stress injury, and there was no significant difference in cellular antioxidant activity between FPs and BPs. The in vivo experiments suggested that FP and BP treatment inhibited malondialdehyde (MDA) levels in a D-galactose-induced oxidation model in mice, and significantly increased antioxidant enzyme activity of superoxide dismutase (SOD), glutathione peroxidase (GSH-Px), catalase (CAT), and the total antioxidant capacity (T-AOC). Mechanistically, FPs and BPs exert their antioxidant activity in distinct ways; FPs ameliorated D-galactose-induced oxidative stress injury partly via the activation of nuclear factor erythroid-2-related factor 2 (Nrf2) signaling pathway, while the BP mechanisms need further study.

## 1. Introduction

Oxidative stress is a destructive state of signaling caused by excessive production of reactive oxygen/nitrogen species (ROS/RNS), resulting in imbalanced redox control between oxidants and antioxidants that favors the oxidants [1,2]. Oxidative stress has been considered the key trigger of many chronic diseases, such as cardiovascular disease, inflammation, diabetes, and cancer [3,4]. Therefore, supplementation with external antioxidants is required to resist oxidative stress and maintain oxidative balance. In this context, natural antioxidants, such as polyphenols, carotenoids, phytosterols, ascorbic acid, and polyunsaturated fatty acids, with safe and efficient antioxidant effects, have attracted considerable interest.

Although antioxidant activity can be assessed in many ways on the basis of different mechanisms, several studies on antioxidant foods and components only use in vitro chemical antioxidant methods to characterize their activities, due to the low cost and easy implementation [5,6,7]. It is necessary to realize that in vitro antioxidant evaluations, such as 2,2′-azinobis-ethylbenzothiazoline-6-sulfonic acid (ABTS) assay, 2,2-diphenyl-1-picrylhydrazyl (DPPH) inhibition, and ferric reducing antioxidant power (FRAP) assay, usually have low biological significance. Antioxidant defense is a complex and multicomponent enzymatic defense system, which typically includes GSH-Px, CAT, SOD, and different exogenous antioxidants that may have selective action on specific antioxidant enzymes [8]. In vitro chemical antioxidant methods have limitations for assessing absorption, bioavailability, distribution, and metabolism [9]. As a result, inconsistent results are often observed between the in vitro antioxidant potential and the in vivo responses of antioxidant foods and components [10,11], indicating that in vitro antioxidant potential is sometimes not reproduced in vivo [12]. In order to obtain an accurate antioxidant capacity and avoid one-sided or even contradictory results of tested antioxidants, a combination of multiple methods, such as cellular antioxidant activity (CAA) assays and animal models, are thus needed to evaluate the antioxidant activity of foods and components.

Olive leaves, which are low-value byproducts generated from olive tree cultivation and olive oil processing, contain a considerable quantity of antioxidant-active substances. Phenolic acids, flavonoids, secoiridoids, hydroxycinnamic acids, simple phenols, and triterpenic acids, which have been shown to be have the potential to scavenge reactive oxygen radicals and resist oxidative stress in the body, are the main sources of the antioxidant properties of olive leaves [13,14]. However, phenolic compounds in plants are usually present in free and bound forms [15,16]. In general, BPs are difficult to directly extract by traditional maceration methods, because they are covalently bound to cell wall structural components (e.g., cellulose, hemicellulose, and pectin) [17]. Nevertheless, numerous in vitro antioxidant assays demonstrated that the insoluble BPs in some plants possess a significantly higher antioxidant capacity compared to FPs [18,19,20]. However, the BPs in olive leaves and their contributions to total phenolics and antioxidant activity have received little attention, as most studies on olive leaves have only emphasized the antioxidant activity of FPs. Furthermore, no investigations have been performed to assess the antioxidant capacity of olive leaves both in vitro and in vivo.

Thus, the objectives of this study were to (i) characterize the composition and determine the content of FPs and BPs in olive leaves; (ii) investigate and compare the antioxidant capacities and possible mechanisms of FPs and BPs in olive leaves using chemical antioxidant methods (DPPH, ABTS, and FRAP), a cellular antioxidant assay, and in vivo animal models. This work will provide a theoretical basis for the application of olive leaves as antioxidants in products.

## 2. Materials and Methods

### 2.1. Plant Materials

Fresh olive leaves of the Nocellara del belice cultivar were obtained from the research garden of Zhejiang Academy of Agricultural Sciences (30.28° N, 120.15° E), Zhejiang Province, China. The mature green leaves were randomly picked from different parts of olive trees (7-year-old) in the middle of April 2022. After harvesting, fresh olive leaves were immediately transported to our lab and dried at 105 °C for 90 min in a hot air oven (DHG-9070A, Jinghong, Shanghai, China). The dried leaves were then ground using a grinder (BJ-200, Baijie, Hangzhou, China), passed through a 60-mesh (250 μm) sieve to obtain a fine powder, and then stored in vacuum-sealed bags at −20 °C until extraction.

### 2.2. Chemicals and Reagents

Trolox, Folin–Ciocalteu reagent, 2,4,6-tris(2-pyridyl)-s-triazine (TPTZ), 2′,7′-dichlorodihydrofluorescein diacetate (DCFH-DA), α,α′-azodiisobutyramidine dihydrochloride (ABAP), and 3-(4,5-dimethylythiazol-2-yl)-2,5-diphenyltetrazolium bromide (MTT) were obtained from Sigma-Aldrich (St. Louis, MO, USA). Reduced glutathione (GSH), SOD, GSH-Px, CAT, MDA, and T-AOC test kits were purchased from Nanjing Jiancheng Bioengineering Institute (Nanjing, China). Other reagents were of analytical grade.

### 2.3. Extraction of Free and Bound Phenolic Fractions from Olive Leaves

The FP and BP fractions of the olive leaves were extracted following a previously reported method [21]. Briefly, 1 g of dry olive leaf powder was extracted using 10 mL of a 70% ethanol solution in an ultrasonic bath for 30 min at 50 °C. The extraction was replicated three times, and the filtrates were combined and served as the FP fraction. Then, the BP fraction was extracted from the residue by alkali extraction. First, the residues were mixed with 40 mL NaOH (2 M), and hydrolyzed at room temperature for 4 h on a shaker under nitrogen. After alkaline hydrolysis, the mixture was acidified to pH 2 with 6 M HCl and centrifuged for 5 min with 5000 rpm. The obtained supernatants were extracted five times with ethyl acetate (1:1, *v*/*v*), then the pooled ethyl acetate extracts were evaporated, dissolved in 10 mL of methanol, and labeled as BP fractions. The extraction yield of FP and BP were 315.3 mg/g dry weight (DW) and 73.9 mg/g DW, respectively. The FP and BP fractions extracted from the olive leaves were stored at −20 °C before analysis.

### 2.4. Identification and Quantification of Free and Bound Antioxidants in Olive Leaves

Chemical profiles in the FP and BP fraction of olive leaves were analyzed by a UPLC-Q-Exactive Orbitrap MS (Ultimate 3000, Thermo Fisher Scientific, San Jose, CA, USA) equipped with an ACQUITY UPLC T3 (2.1 × 100 mm, 1.8 µm, Waters, Milford, MA, USA) column. The UPLC-Q-Exactive Orbitrap MS analysis, including gradient elution conditions and mass spectrometer parameters, were described in our previous study [14]. The identification of FPs and BPs in olive leaves was carried out by matching their high-accuracy quasi-molecular ions and ion fragmentation patterns with the built-in database.

The quantification of individual phenolics (i.e., simple phenols, phenolic acids, flavonoids, iridoids, and triterpenic acids) identified by the UPLC-Q-Exactive Orbitrap-MS were analyzed by using a HPLC system (LC-2030C, Shimazu, Kyoto, Japan) equipped with a Diode Array Detector (DAD). The detection wavelengths were 210, 280, and 320 nm [14,22]. An external standard method was used, and the standards hydroxytyrosol, rutin, oleuropein, luteolin, kaempferol, luteolin-7-*O*-glucoside, rhoifolin, apigenin-7-*O*-glucoside, oxalic, maslinic, asiatic, oleanolic, corosolic, ursolic, ferulic, sinapinic, 4-coumaric, caffeic, and chlorogenic acid were used for calculation of calibration curves (Table S1). The results were expressed as μg/g DW.

### 2.5. Determination of TPC and TFC

The TPC and TFC were determined according to the Folin–Ciocalteu and aluminum chloride colorimetric methods, respectively. The specifics of the method are described in detail in our previous research [14].

### 2.6. Chemical Antioxidant Activity Evaluation

The chemical antioxidant activity of the FP and BP fractions from olive leaves were determined using DPPH, ABTS, and FRAP assays according to previously reported methods [23], and the absorbances at 517 nm, 734 nm, and 593 nm were measured using a spectrophotometer (UV-2600, UNICO, Shanghai, China). Trolox was used as the control [24], and the results were expressed as mg Trolox equivalent/gram of dry olive leaves (μmol TE/g DW).

### 2.7. Cellular Antioxidant Activity Evaluation

#### 2.7.1. Cell Culture and Cytotoxicity Assay

The HepG2 cell at passages 5–10 was obtained from Beijing Dingguo Changsheng Biotechnology Co., Ltd. (Beijing, China). HepG2 cells were cultured in DMEM medium containing 10% fetal bovine serum, 100 U/mL penicillin, and 100 μg/mL streptomycin in a 5% CO_2_ incubator at 37 °C. Cytotoxicity was measured using MTT assay, with slight modifications based on a previous study [25]. Briefly, 190 μL of HepG2 cells were seeded in 96-well plates at a density of 1 × 10^5^ cells/mL, and 10 μL of different concentrations (0, 5, 10, 20, 50, 100, 200, 250, and 500 μg/mL) of olive leaf FP or BP extracts were added to the cells for 24 h. After that, an MTT solution (5 mg/mL) was added to each well, and the absorbance values were measured at 490 nm.

#### 2.7.2. H_2_O_2_-Induced Oxidative Stress in HepG2 Cells

The cells were inoculated into 96-well plates (190 μL) at a density of 1 × 10^5^ cells/mL for 24 h, and 10 μL of H_2_O_2_ (0, 100, 200, 400, 800, 1200, 1600, and 3200 μmol/L) were added to the cells for 1, 2, 4, 6, and 8 h, respectively. Then, the cell survival rate was measured by the MTT assay to screen the optimal H_2_O_2_ treatment concentration and incubation time for constructing the oxidative stress HepG2 cell model. As shown in Tables S2 and S3, the optimal concentration and incubation time for H_2_O_2_ treatment was found to be 800 μmol/L for 6 h according to the half-inhibitory concentration of the cells [26].

HepG2 cells seeded in 96-well plates were incubated for 24 h, and then the cells were grouped as follows: (i) normal group, cells treated with medium but without H_2_O_2_ treatment; (ii) experimental group, cells treated with olive leaf FP or BP extracts and H_2_O_2_ treatment; and (iii) damage group, cells treated with medium and H_2_O_2_ treatment. After the cells were pretreated with 10 μL of different concentrations (20, 50, 100, 200, 250, and 500 μg/mL) of olive leaf FP or BP extracts for 24 h, the medium was removed and 190 μL of fresh medium and 10 μL of 800 μmol/L H_2_O_2_ were added to each well for 6 h of incubation. Finally, the cell survival rate was measured by the MTT assay as described above.

#### 2.7.3. CAA Assay

The CAA of the FP and BP fractions in the olive leaves were determined as described by Wolfe and Liu [27]. HepG2 cells were plated with a density of 6 × 10^4^ cells/well in 96-well black microplates and incubated for 24 h; after that, the growth medium was removed and the cells were washed with PBS to remove the non-adherent and dead cells. Then, the cells were cultivated for 1 h with 100 µL of FP and BP extracts or quercetin with 25 µM DCFH-DA dissolved in medium. After that, the culture medium was removed, and HBSS solution (with 600 µM ABAP) was added to each well. Finally, the black microplates were placed into a fluorescent microplate reader (Spectra MAX 190, Molecular Devices, San Jose, CA, USA). The real-time fluorescence was read every 5 min for 1 h with an excitation wavelength of 485 nm and an emission wavelength of 538 nm.

### 2.8. In Vivo Antioxidant Activity Evaluation

#### 2.8.1. Animals and Experimental Design

Male ICR mice (six weeks, 20 ± 2 g) were purchased from Hangsi Laboratory Animal Co., Ltd. (Hangzhou, China). All animal procedures were conducted in compliance with institutional guidelines for the care and use of laboratory animals at the Zhejiang Academy of Agricultural Sciences (Certificate NO.2023ZAASLA80). After 7 days of adaptive feeding, the mice were randomly divided into seven groups (*n* = 8): (1) control group (administrated 0.1 mL/10 g body weight (b.w.) saline); (2) model group (administrated 0.1 mL/10 g b.w. saline); (3) Vitamin C (Vc) group (administrated 100 mg/kg b.w. Vc); (4) FP-L (administrated 125 mg/kg b.w. FPs); (5) FP-H (administrated 250 mg/kg b.w. FPs); (6) BP-L (administrated 125 mg/kg b.w. BPs); and (7) BP-H (administrated 250 mg/kg b.w. BPs).

**Figure 1:**
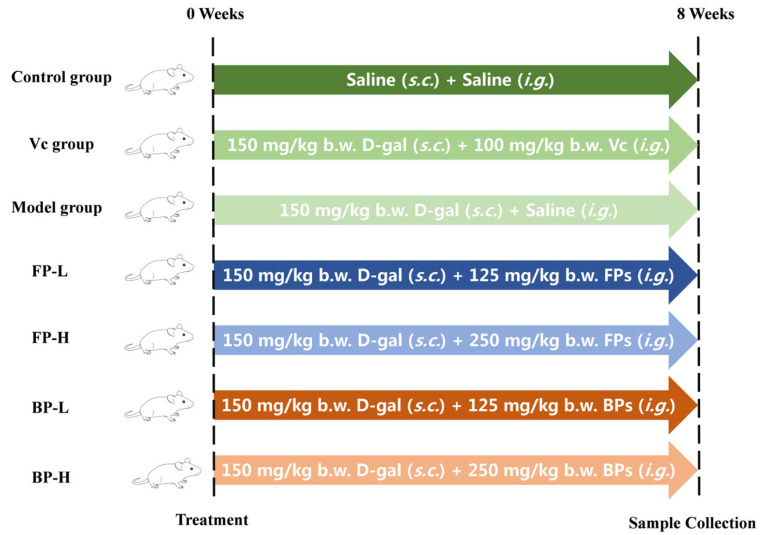
The schematic diagram of the experimental design for evaluating the in vivo antioxidant ability of FPs and BPs in olive leaves.

All solutions were administered once daily by gastric gavage for 8 weeks. In addition, all mice except those in the control group received subcutaneous injections of D-galactose at a dose of 150 mg/kg b.w. daily, and the control group received injections of saline (Figure 1). After 8 weeks, mice were fasted for 12 h before being euthanized with CO_2_ and sacrificed by cervical dislocation to obtain blood samples and heart, kidney, and liver tissues.

#### 2.8.2. Histological Analysis

The fixed liver tissues were embedded in paraffin, then sectioned (5-mm thick) and stained with hematoxylin and eosin (H&E) for histopathology. After installation, the slides were viewed under a microscope (DXIT 1200, Nikon, Tokyo, Japan).

#### 2.8.3. Biochemical Assays

The in vivo antioxidant activity in the serum, liver, and kidney was evaluated by measuring MDA, GSH-Px, SOD, CAT, GSH, and T-AOC using commercial ELISA kits according to the instructions.

#### 2.8.4. RT-qPCR Analysis

Hepatic total RNA was isolated using Trizol reagent, followed by reverse transcription of total RNA to cDNA. The mRNA expression levels were measured by an RT-qPCR system (ABI PRISM 7300, Applied Biosystems, Foster City, CA, USA), and the results were calculated by the 2^−ΔΔCT^ method. GAPDH was used as an internal reference control, and primer sequences used in this study are shown in Table S4.

### 2.9. Statistical Analysis

All results are expressed as mean ± SD. The data were processed by one-way ANOVA, followed by Tukey’s post hoc tests using SPSS Statistics software (V21.0, SPSS Inc., Chicago, IL, USA), with the significant difference set at *p* < 0.05. Origin software (2019b, Originlab Inc., Massachusetts, MA, USA) was used for EC_50_ analysis.

## 3. Results and Discussion

### 3.1. Identification and Quantification of Free and Bound Antioxidants in Olive Leaves

Phenolic compounds in plants exist in free and bound forms [28]. Although BPs are usually found at lower levels compared to FPs, numerous studies have demonstrated that BPs in some food matrices possess a significantly higher antioxidant capacity than FPs [18,19]. Previously, the profiles of BPs in olive leaves and their contributions to antioxidant activity were not studied. Therefore, the chemical profiles of FP and BP fractions in olive leaves were investigated in this research, and their in vitro and in vivo antioxidant capacity and possible mechanism were studied comprehensively.

The chemical profiles of FP and BP fractions in olive leaves were preliminarily identified by using a UPLC-Q-Exactive Orbitrap-MS, and the identified 28 compounds along with their retention time (Rt), mass data, MS/MS fragments, and molecular formula are described in Table 1 and Figure S1. These compounds were classified into five different families according to their structural skeletons, and included one simple phenol, five phenolic acids, fifteen flavonoids, two iridoids, and five triterpenoid acids. As shown in Table 1, 24 compounds were identified in the FP fractions of olive leaves, with flavonoids, iridoids, and triterpenic acids being the primary class. The chemical profiles of FP fractions are in accordance with previous findings; Contreras et al. [29] and Dias et al. [30] analyzed FP fractions of olive leaves by liquid chromatography coupled to mass spectrometry techniques and also revealed their richness in flavonoids (luteolin, quercetin, rutin, luteolin-7-*O*-glucoside, etc), iridoids (oleuropein and secoxyloganin), and simple phenols (hydroxytyrosol). In addition, a total of 14 phenolics were detected in the BP fractions, consisting of flavonoids (6), phenolic acids (5), triterpenic acids (2), and simple phenol (1). As far as we know, this is the first time that phytochemical profiles of BP fractions were identified in olive leaves.

The individual phenolic compounds were further quantified, and our results demonstrated that the FP and BP fractions had different phenolic profiles, as indicated in Table 2. The highest phenolic content detected in the FP fraction was that of oleuropein (17.52 mg/g DW), followed by luteolin-7-*O*-glucoside (2.80 mg/g DW) and kaempferol-7-*O*-glucoside (2.07 mg/g DW), which was consistent with the previously reported findings of the FP composition in olive leaves [14]. In the BP fraction, the content of sinapinic acid was highest at 0.39 mg/g DW, followed by apigenin-7-*O*-neohesperidoside (0.31 mg/g DW), 4-coumaric acid (0.21 mg/g DW), ferulic acid (0.20 mg/g DW), hydroxytyrosol (0.19 mg/g DW), and caffeic acid (0.16 mg/g DW). It was noteworthy that higher levels of phenolic acid (i.e., sinapinic acid, 4-coumaric acid, ferulic acid, and caffeic acid) and hydroxytyrosol were detected in the BP fraction; however, flavonoids, triterpenoid acids, and iridoids were more concentrated in the free form.

Oleuropein, secoxyloganin, maslinic acid, and glycosylated flavonoids are usually the most abundant phenolic compound in olive leaves, and are easily and efficiently extracted by organic aqueous mixtures [14]. However, phenolic acids are mostly covalently linked to structural components of the cell walls forming cross-links in plants, and thus cannot be easily extracted directly by solvent extraction; this may explain why phenolic acids are typically found in bound forms, with free forms being extremely rare [31,32]. Most phenolic acids, such as gallic, *p*-coumaric, ferulic, and caffeic acid, are also found at higher levels in their bound forms than in their corresponding free forms in sorghum and mango leaves [19,31]. Phenolic acids, with many phenolic hydroxyl groups in the molecules, are excellent hydrogen or electron donors that quench free radicals [33,34]. Thus, phenolic acids are probably important contributors to the antioxidant capacity of the BP fraction in olive leaves. Further research aims to release FPs in olive leaves through food processing, such as ultra-high pressure, alkaline or enzymatic hydrolysis, and fermentation.

### 3.2. Chemical Antioxidant Activities of FP and BP Fractions in Olive Leaves

Chemical antioxidant methods are widely used for preliminary screening of oxidation/reduction potentials of antioxidants due to their low cost and easy implementation. TPC and TFC were found to be directly associated with chemical antioxidant activities [6,23]. In this study, the TPC and TFC of the FP and BP fractions of olive leaves were evaluated prior to the chemical antioxidant assay. As shown in Table 3, the TPC and TFC levels in the FP fraction were statistically higher than those in the BP fraction (26.39 vs. 0.65 mg GAE/g DW for TPC, and 145.30 vs. 1.73 mg RE/g DW for TFC, *p* < 0.05). The bound phenolics contributed to only 2.4% of the total phenolics content, suggesting that phenolic compounds in olive leaves were largely present in a free form. The result was in agreement with previous findings reporting that most phenolics in vegetables and fruits were found in free forms, and those in insoluble bound forms generally accounted for no more than 24% of the total phenolic content [17,35].

Furthermore, three chemical methods, including DPPH and ABTS for evaluating the scavenging ability of radicals and the FRAP method for evaluating the iron reduction capacity, were used to estimate the antioxidant activities of FP and BP fractions in olive leaves. As shown in Table 3, the DPPH and ABTS+. free radical scavenging ability of the FP fraction was significantly higher than that of the BP fraction, with values of 26.09 vs. 10.44 μmol TE/g DW with the DPPH assay, and 249.49 vs. 6.66 μmol TE/g DW with the ABTS assay. The FRAP assay also showed a significant order of magnitude difference between the FPs and BPs, with values of 13.79 vs. 0.37 μmol TE/g DW, respectively. The presence of rich polyphenols, such as flavonoids, triterpenoid acids, and particularly oleuropein, of which the levels were very high, could be the reason for the high DPPH and ABTS+. radical-scavenging property of the FP fraction [36]. In conclusion, the chemical antioxidant activity indicated that the BP fraction in olive leaves had a weak DPPH and ABTS+. radical-scavenging ability.

### 3.3. Cellular Antioxidant Activities of FP and BP Fractions in Olive Leaves

Currently, chemical antioxidant activity assays have a major role in testing antioxidant effects; however, their ability to predict in vivo activity has been questioned due to the limitations on absorption, bioavailability, distribution, and metabolism assessment [9]. Therefore, we established a cellular antioxidant system to compare and investigate the difference between the biological and chemical antioxidant ability of the FP and BP fractions in olive leaves in this study.

#### 3.3.1. FP and BP Fractions in Olive Leaves Resist H_2_O_2_-Induced Oxidative Stress in HepG2 Cells

**Figure 2:**
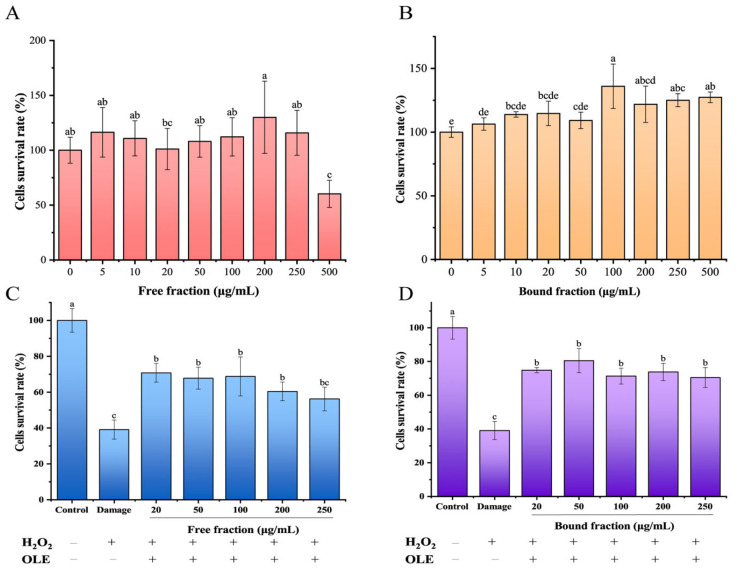
Protective effect of FPs and BPs in olive leaves against H_2_O_2_-induced oxidative stress in HepG2 cells. (**A**,**B**) The effect of FPs and BPs on the cell survival rate of HepG2 cells. (**C**,**D**) The effect of FPs and BPs on the cell survival rate of HepG2 cells induced by H_2_O_2_. Different letters indicate significant differences (*p* < 0.05).

First, the cytotoxicity of FPs and BPs in HepG2 cells was investigated to screen for a concentration that did not exhibit significant toxicity in cells. As shown in Figure 2A, FPs up to 250 μg/mL did not impact cell viability, but at 500 μg/mL, the cell survival rate decreased to 60.39 ± 12.17%. The BPs at concentrations of 5–500 μg/mL were nontoxic to HepG2 cells, with the cell survival rate between 106.35% and 136.01% (Figure 2B). The subsequent concentrations of FPs and BPs for cellular antioxidant activity assays were set as 0–250 μg/mL.

H_2_O_2_ is a well-recognized oxidative stress inducer, which can penetrate the cell membrane and generate excessive ROS, leading to oxidative stress and eventually injuring the cell [37]. Next, the protection of FPs and BPs against H_2_O_2_-induced oxidative stress was evaluated in the study. As presented in Figure 2C,D, the cell viability of the H_2_O_2_-damaged group was remarkably decreased to 39.10% in comparison with the normal group (*p* < 0.05). Encouragingly, pretreatment with both the FPs and BPs effectively inhibited the decrease in H_2_O_2_-induced cell viability. The cell viability of HepG2 cells was 70.75% and 74.81% with pretreatment of FPs and BPs at 20 mg/mL, respectively, and was almost unchanged as the FP and BP concentration increased from 20 to 250 μg/mL. The protective capacities of FP and BP on H_2_O_2_-induced oxidative stress are not surprising and can be associated with the presence of phenolic compounds. For example, Goncalves et al. [38] reported phenolic-enriched fractions from sweet cherries had a significant ability to prevent HepG2 cells against H_2_O_2_-induced oxidative injury. Furthermore, many studies have reported that hydroethanolic extracts from vegetal leaf parts were rich in phenolic and flavonoid compounds (e.g., *Apios americana* Medik, *Vaccinium dunalianum* Wight and *Hemerocallis fulva*), and also showed strong ability to inhibit the H_2_O_2_-induced cells damage [39,40,41]. The results in this study suggested that both FPs and BPs in olive leaves protected HepG2 cells from H_2_O_2_-induced oxidative stress injury, and that BPs showed better antioxidant activity for rescuing H_2_O_2_-induced cell viability losses than did FPs.

#### 3.3.2. FP and BP Fractions in Olive Leaves Exhibited Cellular Antioxidant Activity

**Figure 3:**
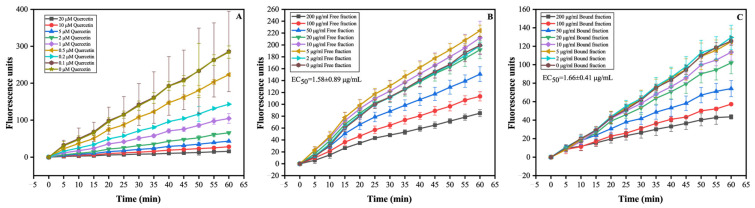
Kinetic curves of peroxyl radical-induced DCF fluorescence and the inhibition of oxidation by quercetin (**A**), FPs (**B**) and BPs (**C**) on the fluorescence in HepG2 cells over time.

The CAA assay, which measures the ability of antioxidants to inhibit the formation of fluorescent DCF by ABAP-generated peroxyl radicals in the HepG2 cell model, was used to assess the biological antioxidant capacity of FPs and BPs in olive leaves. As presented in Figure 3, the fluorescence from DCF formation in HepG2 cells decreased with increased quercetin, FP, and BP concentrations, suggesting that they have great antioxidant capacity. The EC_50_ values of CAA for FPs and BPs was 1.58 μg/mL and 1.66 μg/mL, respectively, suggesting that there was no significant difference in the CAA value between the FP and BP fraction (*p* > 0.05). The observations on CAA were inconsistent with the conclusion by the chemical antioxidant evaluation, which found that the BP fraction had a weaker ability to eliminate DPPH and ABTS+. free radicals, and a significant order of magnitude difference was found between FPs and BPs. Perhaps this difference is related to the higher content of phenolic acids (i.e., sinapinic acid, ferulic acid, and caffeic acid) in BPs, which can increase the activity of antioxidant enzymes, such as CAT, GSH-Px, SOD, and heme oxygenase-1 (HO-1), and thereby activate the intracellular antioxidant defense system [12]. The contradictory conclusions between the chemical antioxidant and cellular antioxidant responses has been previously observed with certain antioxidant foods and components, such as inulin, tangeretin, and citrus flavanones [11,42]. The cell-based antioxidant assay is more biologically relevant than chemical tests due to its sensitivity for cell uptake, metabolism, and intracellular location [9]. Thus, antioxidant assays chosen in future studies should be further extended to obtain a more complete panorama of the antioxidant activity, and should not be limited to an in vitro chemical antioxidant assessment.

### 3.4. In Vivo Antioxidant Activities of FP and BP Fractions in Olive Leaves

**Figure 4:**
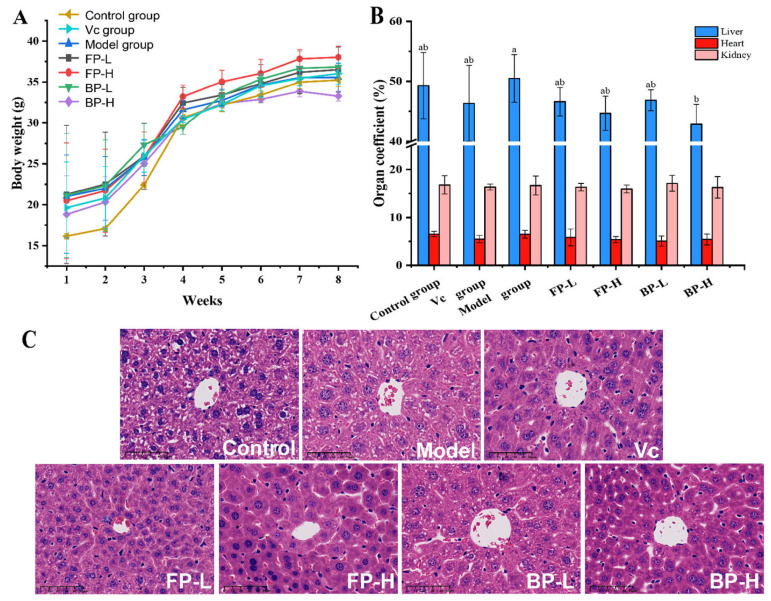
Effects of FPs and BPs in olive leaves on body weight (**A**), organ coefficients (**B**), and H&E staining (**C**) in D-galactose-induced aging mice. Scale bar = 50 μm. Different letters indicate significant differences (*p* < 0.05).

D-galactose is widely used for establishing an oxidation model in mice [43]. Excessive D-galactose in the body will produce aldose, hydrogen peroxide, and other oxygen free radicals, causing the accumulation of reactive oxygen species, and ultimately leading to oxidative stress [44]. In this research, D-galactose was chosen to establish the oxidation model for evaluating the in vivo antioxidant capacity and possible mechanisms of FPs and BPs in olive leaves (Figure 1). As shown in Figure 4A, the body weight of mice in all groups exhibited an upward trend without differences among groups during the 8-week experiments. The index results of the kidney, liver, and heart demonstrated that D-galactose injections slightly decreased the organ indexes of mice without statistically significant differences (Figure 4B), which was consistent with previous reports [45].

#### 3.4.1. FP and BP Fractions in Olive Leaves Protect Liver Tissue in Aging Mice

The liver is highly sensitivity to the oxidative damage caused by D-galactose [46]. According to the hepatic H&E staining results (Figure 4C), the liver structure of mice in the control group had a normal cell morphology and was neatly arranged around the central vein, with prominent nuclei and abundant cytoplasm with a uniform distribution. Compared with the control group, the model group exhibited apparent liver damage, which was characterized by vacuolar degeneration, necrosis, and structural disorder, indicating that the D-galactose injections induced liver damage in mice. Treatment with Vc, FPs, and BPs significantly ameliorated liver damage, as evidenced by cells arranged in a more orderly manner, and the structures tended to be complete, indicating the protective effect of FPs and BPs on liver tissue.

#### 3.4.2. FP and BP Fractions in Olive Leaves Relieve Oxidative Stress in Aging Mice

**Figure 5:**
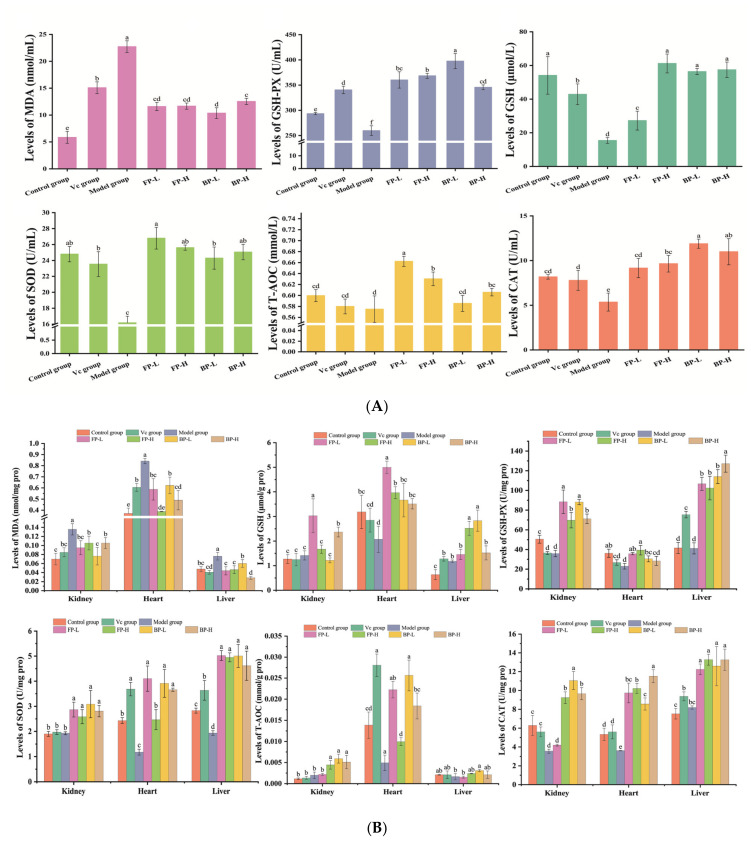
Effect of FPs and BPs in olive leaves on antioxidant properties in D-galactose-induced aging mice: (**A**) serum; and (**B**) kidney, heart, and liver tissues. Different letters indicate significant differences (*p* < 0.05).

The body has several natural defense systems to counteract oxidative stress, including the production of antioxidant enzymes (SOD, CAT, and GSH-Px) and the action of non-enzymatic antioxidants, such as GSH and Vitamin E [47]. However, endogenous and exogenous oxidants disrupt this antioxidant system, causing accumulation of reactive oxygen species and producing peroxidation products, and ultimately causing oxidative stress [48]. MDA is one of the products of lipid peroxidation, which can reflect the severity of a free radical attack on cells [49]. As shown in Figure 5, the mice in the model group that received subcutaneous injections of D-galactose for 8 weeks displayed 3.9-fold, 2.2-fold, 2.3-fold, and 1.7-fold higher MDA levels in the serum than those of the control group, kidney, heart, and liver, respectively. The FP and BP extract treatments significantly reversed the increased MDA content compared with the model group, indicating that FP and BP extracts effectively regulated the redox status in D-galactose-induced aging mice. Furthermore, the antioxidant enzymes (SOD, CAT, and GSH-Px), the T-AOC, and the non-enzymatic GSH levels in the serum, kidney, heart, and liver of mice were measured to evaluate the repair effect of FPs and BPs on oxidative stress in aging mice. As shown in Figure 5A,B, the levels of CAT, SOD, and GSH-Px in the serum, kidney, and heart of mice in the model group were lower than those in the control group (*p* < 0.05). Each dose of FP and BP increased the activities of SOD, GSH-Px, and CAT in aging mice without a dose-response relationship. In addition, we observed that both FPs and BPs effectively improved the T-AOC and the non-enzymatic GSH level in aging mice. In conclusion, the data from this work showed that the FP and BP fractions were effective in mitigating oxidative damage by restoring SOD, CAT, and GSH-Px activities, and the content of MDA in aging mice.

#### 3.4.3. FP Fractions in Olive Leaves Activate Nrf2 Signaling in Aging Mice

**Figure 6:**
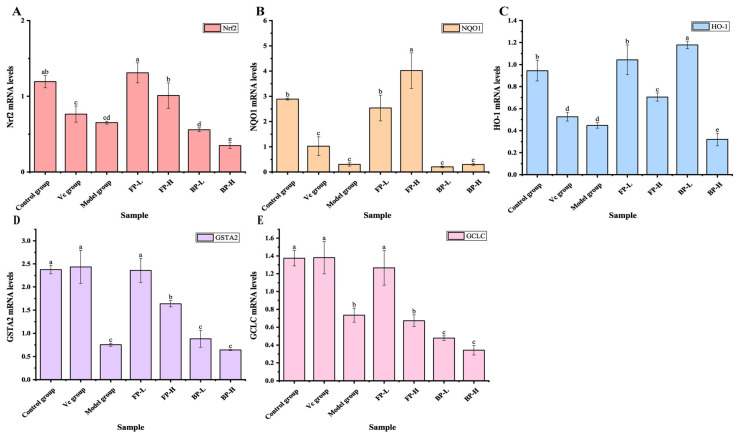
Effect of FPs and BPs in olive leaves on the expression of Nrf2 signaling in D-galactose-induced aging mice: (**A**) Nrf2; (**B**) NQO1; (**C**) HO-1; (**D**) GSTA2; and (**E**) GCLC. Different letters indicate significant differences (*p* < 0.05).

Nrf2 has been recognized as a key antioxidant transcription factor involved in redox homeostasis by targeting many antioxidant genes, including NAD(P)H dehydrogenase quinone 1 (NQO1), HO-1, glutamate-cysteine ligase catalytic subunit (GCLC), CAT, and SOD [50,51]. Thus, the expression levels of Nrf2, HO-1, NQO1, GCLC, and Glutathione S-transferase alpha 2(GSTA2) were determined in this research. The results showed that D-galactose administration decreased the expression of Nrf2 compared to the control group (Figure 6A), which was consistent with a previous study that reported that the D-galactose treatment inhibited the translocation of Nrf2 [45]. FP treatment reversed the effect of D-galactose on the expression of Nrf2 in mice; in particular, 125 mg/kg of FPs resulted in a 2.84-fold increase in the expression of Nrf2 compared to the model group. This result indicated that there was not a dose-response relationship between Nrf2 expression and the FP concentration. In contrast, the BP-treated groups, both at 125 and 250 mg/kg, did not affect the expression of Nrf2. As shown in Figure 6B–E, the increase of Nrf2 in the FP-treated group eventually led to the upregulation of the targeted genes HO-1, NQO1, GCLC, and GSTA2 in mouse livers, while BP-treated groups did not have increased expression of these genes. This shows that even though both FPs and BPs effectively increased antioxidant enzyme activity and prevented oxidative damage in vivo, there was a great difference in their mechanisms. Perhaps this difference is related to the different chemical profiles of FPs and BPs, with the polyphenols in FPs protecting against oxidative stress by elevating gene expression of Nrf2. FPs ameliorate D-galactose-induced oxidative stress injury partly via the activation of the Nrf2 signaling pathway, while the mechanism of BPs need further study.

## 4. Conclusions

**Figure 7:**
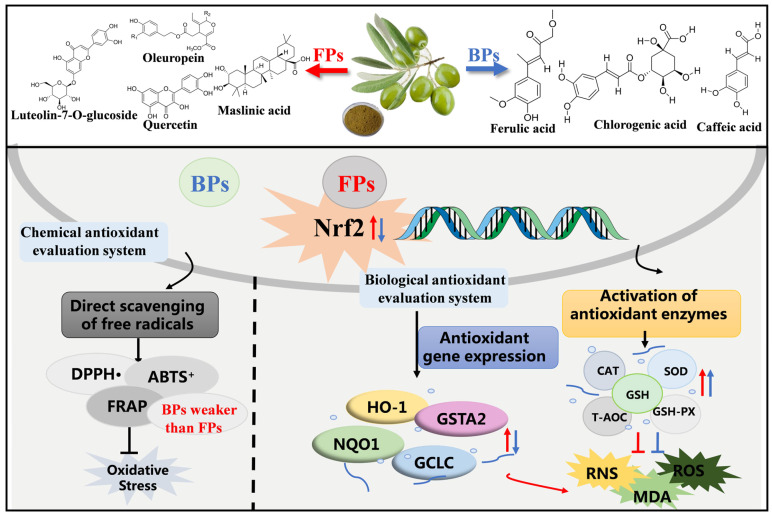
Potential molecular mechanisms of antioxidant properties by FPs and BPs in olive leaves. The red arrows represent FPs, and the blue arrows represent BPs.

The present study investigated and compared the chemical profiles, contents, and in vitro and in vivo antioxidant capacities of FPs and BPs in olive leaves. The results showed that FPs and BPs exhibited different antioxidant effects in the chemical antioxidant evaluation systems compared to the biological antioxidant evaluation systems. The chemical antioxidant evaluation systems (DPPH, ABTS, and FRAP assays) indicated that FPs exerted significantly higher antioxidant activity than did the BP fraction, and BPs had a weak ability to directly scavenging DPPH and ABTS+. free radicals. In biological antioxidant evaluation systems (cellular and in vivo mouse models), both FPs and BPs exhibited similar antioxidant effects by effectively protecting HepG2 cells from H_2_O_2_-induced oxidative stress injury, and were effective in mitigating oxidative damage by restoring SOD, CAT, and GSH-Px activities in aging mice. Further exploration showed that FPs ameliorated D-gal-induced oxidative stress injury partly via the activation of the Nrf2 signaling pathway, while the mechanism of BPs requires further study (Figure 7). The different manner by which FPs and BPs exert antioxidant activity is probably related to the different chemical profiles of FPs and BPs. In conclusion, it could be stated that the in vitro chemical antioxidant assessment of antioxidant foods and components is not sufficient to estimate their antioxidant effects in vivo. To obtain a more complete panorama of the antioxidant activity, the antioxidant assay chosen should be further extended to cellular and/or in vivo animal systems in future studies.

## Figures and Tables

**Table 1:** Compounds identified by UPLC-Q-Exactive Orbitrap-MS in free and bound phenolic fraction of olive leaves.

No	Rt (min)	Measured *m*/*z*	MS/MS Fragments	Molecular Formula	CAS	Compounds	Class	FP	BP
1	3.912	153.0550 [M − H]^−^	123.04, 153.05	C8H10O3	10597-60-1	Hydroxytyrosol	Simple phenols	X	X
2	5.296	353.0880 [M − H]^−^	191.06, 209.66	C_16_H_18_O_9_	327-97-9	Chlorogenic acid	Phenolic acids	X	X
3	5.790	163.0390 [M + H]^+^	163.04, 145.03, 135.04	C_9_H_8_O_4_	331-39-5	Caffeic acid	Phenolic acids		X
4	6.605	147.0440 [M + H]^+^	147.04, 119.05	C_9_H_8_O_3_	7400-08-0	4-Coumaric acid	Phenolic acids		X
5	7.110	223.0610 [M − H]^−^	164.05, 208.04, 223.06	C_11_H_12_O_5_	530-59-6	Sinapinic acid	Phenolic acids		X
6	7.155	177.0646 [M + H]^+^	177.05, 145.03	C_10_H_10_O_4_	537-98-4	Ferulic acid	Phenolic acids		X
7	5.698	403.1250 [M − H]^−^	59.01, 71.01, 89.02	C17H24O11	58822-47-2	Secoxyloganin	Iridoids	X	
8	7.525	539.1776 [M-H]^−^	539.18, 377.12, 275.09	C_25_H_32_O_13_	32619-42-4	Oleuropein	Iridoids	X	
9	5.916	609.1469 [M − H]^−^	447.09, 609.15, 285.04	C_27_H_30_O_16_	52187-80-1	Luteolin-3′,7-di-*O*-glucoside	Flavonoids	X	
10	6.523	609.1470 [M − H]^−^	609.15, 300.03, 301.04	C_27_H_30_O_16_	153-18-4	Rutin	Flavonoids	X	X
11	6.528	611.1608 [M + H]^+^	303.05, 466.11, 85.03	C_27_H_30_O_16_	18016-58-5	Quercetin 3-*O*-glucoside-7-*O*-rhamnoside	Flavonoids	X	
12	6.753	465.1026 [M + H]^+^	303.05, 85.03	C_21_H_20_O_12_	482-35-9	Quercetin-3β-D-glucoside	Flavonoids	X	
13	6.758	449.1077 [M + H]^+^	449.11, 287.05	C_21_H_20_O_11_	5373/11/5	Luteolin-7-*O*-glucoside	Flavonoids	X	X
14	6.941	577.1570 [M − H]^−^	269.05, 577.16	C_27_H_30_O_14_	17306-46-6	Apigenin-7-*O*-neohesperidoside	Flavonoids	X	X
15	7.143	609.1815 [M + H]^+^	301.07, 609.18	C_28_H_32_O_15_	38665-01-9	Neodiosmin	Flavonoids	X	
16	7.214	477.1043 [M − H]^−^	431.10, 268.04, 269.05	C_21_H_20_O_10_	578-74-5	Apigenin-7-*O*-glucoside	Flavonoids	X	X
17	7.236	447.0937 [M − H]^−^	285.04, 447.09	C_21_H_20_O_11_	16290-07-6	Kaempferol-7-*O*-glucoside	Flavonoids	X	X
18	7.390	447.0939 [M− H]^−^	210.04, 285.04	C21H20O11	6920-38-3	Luteolin-4′-*O*-glucoside	Flavonoids	X	
19	8.919	285.0407 [M − H]^−^	285.04, 299.46	C_15_H_10_O_6_	491-70-3	Luteolin	Flavonoids	X	X
20	8.922	287.0548 [M + H]^+^	287.05, 269.08	C_15_H_10_O_6_	520-18-3	Kaempferol	Flavonoids	X	
21	9.012	303.0496 [M − H]^−^	303.05, 285.15	C_15_H_10_O_7_	117-39-5	Quercetin	Flavonoids	X	
22	9.882	301.0703 [M + H]^+^	301.07, 286.05	C_16_H_12_O_6_	520-34-3	Diosmetin	Flavonoids	X	
23	10.001	315.0514 [M − H]^−^	315.05, 300.03	C_16_H_12_O_7_	1486-70-0	3-*O*-Methylquercetin	Flavonoids	X	
24	11.579	487.3434 [M − H]^−^	487.34, 469.33	C30H_48_O_5_	464-92-6	Asiatic acid	Triterpenoid acids	X	
25	13.414	471.3483 [M − H]^−^	471.35, 428.24	C_30_H_48_O_4_	4373-41-5	Maslinic acid	Triterpenoid acids	X	
26	13.434	455.3517 [M + H]^+^	205.16, 189.16, 203.18	C_30_H_48_O_4_	4547-24-4	Corosolic acid	Triterpenoid acids	X	X
27	12.759	457.3673 [M + H]^+^	457.37, 203.18, 191.18	C_30_H_48_O_3_	508-02-1	Oleanolic acid	Triterpenoid acids	X	
28	16.492	439.3567 [M + H]^+^	411.36, 439.36, 203.18	C_30_H_48_O_3_	77-52-1	Ursolic acid	Triterpenoid acids	X	X

X: presence of the compound. FP and BP represent free phenolic and bound phenolic, respectively.

**Table 2:** Quantification of phenolics compounds in free and bound phenolic fraction of olive leaves.

No.	Compounds	Content (mg/g DW)		No.	Compounds	Content (mg/g DW)	
		FP Fraction	BP Fraction			FP Fraction	BP Fraction
1	Hydroxytyrosol	0.11 ± 0.01 ^b^	0.19 ± 0.09 ^a^	15	Apigenin-7-*O*-glucoside	0.47 ± 0.03 ^a^	0.05 ± 0.01 ^b^
2	Chlorogenic acid	0.13 ± 0.02 ^a^	0.07 ± 0.01 ^b^	16	Neodiosmin	0.54 ± 0.03	ND
3	Caffeic acid	ND	0.16 ± 0.01	17	Kaempferol-7-*O*-glucoside	2.07 ± 0.15 ^a^	0.14 ± 0.03 ^b^
4	4-Coumaric acid	ND	0.21 ± 0.03	18	Luteolin-4′-*O*-glucoside	0.61 ± 0.04	ND
5	Sinapinic acid	ND	0.39 ± 0.03	19	Luteolin	0.38 ± 0.03 ^a^	0.03 ± 0.01 ^b^
6	Ferulic acid	ND	0.20 ± 0.08	20	Kaempferol	0.03 ± 0.01	ND
7	Secoxyloganin	2.11 ± 0.18	ND	21	Quercetin	0.12 ± 0.01	ND
8	Oleuropein	17.52 ± 2.61	ND	22	Diosmetin	0.03 ± 0.00	ND
9	Luteolin-3′,7-di-*O*-glucoside	0.44 ± 0.03	ND	23	3-*O*-Methylquercetin	0.02 ± 0.00	ND
10	Rutin	1.31 ± 0.10 ^a^	0.12 ± 0.01 ^b^	24	Asiatic acid	0.26 ± 0.01 ^a^	ND
11	Quercetin 3-*O*-glucoside-7-*O*-rhamnoside	0.52 ± 0.03	ND	25	Maslinic acid	1.31 ± 0.02 ^a^	0.01 ± 0.00 ^b^
12	Quercetin-3β-D-glucoside	0.78 ± 0.06	ND	26	Corosolic acid	0.39 ± 0.08	ND
13	Luteolin-7-*O*-glucoside	2.80 ± 0.27 ^a^	0.12 ± 0.04 ^b^	27	Oleanolic acid	1.61 ± 0.07 ^a^	0.06 ± 0.00 ^b^
14	Apigenin-7-*O*-neohesperidoside	0.71 ± 0.04 ^a^	0.31 ± 0.02 ^b^	28	Ursolic acid	ND	0.02 ± 0.01

Means followed by different letters (^a^, ^b^) are significant differences (*p* < 0.05); FP and BP represent free phenolic and bound phenolic, respectively; ND, not detected.

**Table 3:** TPC, TFC and the chemical antioxidant activity of free and bound phenolic fraction from olive leaves.

	TPC (mg GAE/g DW)	TFC (mg RE/g DW)	DPPH (μmol TE/gDW)	ABTS (μmol TE/gDW)	FRAP (μmol TE/gDW)
FP fraction	26.39 ± 1.26 ^a^	145.30 ± 9.11 ^a^	26.09 ± 0.03 ^a^	249.49 ± 0.29 ^a^	13.79 ± 0.03 ^a^
BP fraction	0.65 ± 0.05 ^b^	1.73 ± 0.29 ^b^	10.44 ± 0.01 ^b^	6.66 ± 0.01 ^b^	0.37 ± 0.00 ^b^

Means followed by different letters (^a^, ^b^) are significant differences (*p* < 0.05); FP and BP represent free phenolic and bound phenolic, respectively.

## Data Availability

Data are contained within the article and Supplementary Materials.

## Supplementary Materials

The following supporting information can be downloaded at: https://www.mdpi.com/article/10.3390/antiox12122033/s1, Figure S1. Total ion chromatogram (TIC) of free and bound phenolics fractions in olive leaves for negative ion and positive ion mode UPLC-Q-Exactive Orbitrap-MS. (A,B) FPs in negative and positive ion mode; (C,D) BPs in negative and positive ion mode. Table S1. Calibration curves used for HPLC quantification. Table S2. Effect of different concentrations of H_2_O_2_ on the viability of HepG2 cells. Table S3. Effect of the incubation time of H_2_O_2_ on the viability of HepG2 cells. Table S4. Primer sequences for RT-qPCR.
