# Supplementary material for: Antioxidant Capacity of Free and Bound Phenolics from Olive Leaves: In Vitro and In Vivo Responses
Source: Antioxidants (Basel). 2023 Nov 23;12(12):2033. doi: 10.3390/antiox12122033 (PMC10740763; doi:10.3390/antiox12122033)
Supplement: Supplementary file 1 [file antioxidants-12-02033-s001.zip › antioxidants-2719742-supplementary.pdf]

Antioxidant Capacity of Free and Bound Phenolics from Olive Leaves: In Vitro and In Vivo Responses

Ting Li [1,2,†], Wenjun Wu [3,†], Jianming Zhang [1], Qinghang Wu [1], Shenlong Zhu [4], Erli Niu [4], Shengfeng Wang [5], Chengying Jiang [3], Daqun Liu [1,*] and Chengcheng Zhang [1,*]

[1] Food Science Institute, Zhejiang Academy of Agricultural Sciences, Hangzhou 310021, China; lt1345600496@126.com (T.L.); zhangjianming@zaas.ac.cn (J.Z.); hang9799@outlook.com (Q.W.)
[2] College of Food and Health, Zhejiang A&F University, Hangzhou 311300, China
[3] Gansu Research Academy of Forestry Science and Technology, Lanzhou 730020, China; wuwenjun121@163.com (W.W.); jcytxb@126.com (C.J.)
[4] Institute of Crop and Nuclear Technology Utilization, Zhejiang Academy of Agricultural Sciences, Hangzhou 310021, China; zhusl@zaas.ac.cn (S.Z.); niuerli@zaas.ac.cn (E.N.)
[5] Research Center of Analysis and Measurement, Zhejiang University of Technology, Hangzhou 310014, China; wsf1027@zjut.edu.cn
* Correspondence: liudaqun@zaas.ac.cn (D.L.); zhangcc@zaas.ac.cn (C.Z.)
† These authors have contributed equally to this work.

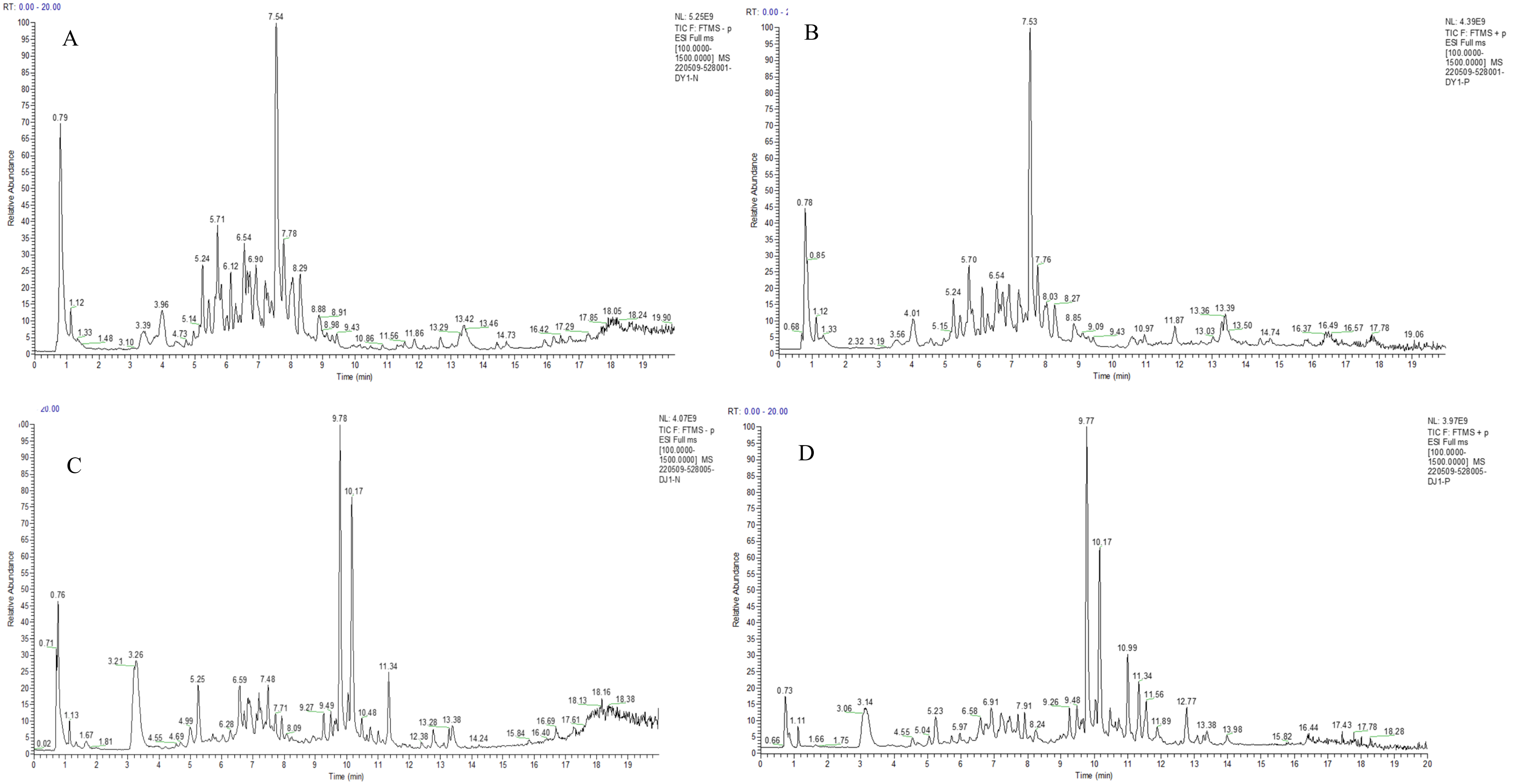

Figure S1. Total ion chromatogram (TIC) of free and bound phenolics fractions in olive leaves for negative ion and positive ion mode UPLC-Q-Exactive Orbitrap-MS. (A-B) FPs in negative and positive ion mode; (C-D) BPs in negative and positive ion mode.

Table S1. Calibration curves used for HPLC quantification.

No.	Calibration curves	R^2	Linear range (μg/mL)	Compounds
1	y = 4389.8x + 2982.5	0.9988	10-200	Asiatic acid
2	y = 3946.9x + 1015.3	0.9992	12-240	Maslinic acid
3	y = 3588.9x + 8597.6	0.9989	10-200	Corosolic acid
4	y = 5122.4x + 2077.2	0.9992	10-200	Oleanolic acid
5	y = 4409.7x + 3699.8	0.9986	10-200	Ursolic acid
6	y = 19133x - 30576	0.9997	10-200	Chlorogenic acid
7	y = 41220x - 584378	0.9890	24-480	Caffeic acid
8	y = 68816x - 581501	0.9901	15-300	4-Coumaric acid
9	y = 12851x - 12745	0.9999	25-500	Sinapinic acid
10	y = 35342x - 75709	0.9994	12-240	Ferulic acid
11	y = 10534x - 1991	0.9997	10-200	Hydroxytyrosol
12	y = 7341.7x - 2253.2	0.9997	10-200	Rutin
13	y = 12430x + 12561	0.9995	10-200	Luteolin-7-O-glucoside
14	y = 11641x - 3972.8	0.9997	10-200	Rhoifolin
15	y = 12855x - 12713	0.9996	10.5-210	Apigenin-7-O-glucoside
16	y = 2334.1x - 1014.7	0.9998	11-220	Oleuropein
17	y = 19079x - 15381	0.9998	10-200	Quercetin
18	y = 13704x - 4919.6	0.9998	10-200	Luteolin
19	y = 16509x - 6454.5	0.9997	11.5-230	Kaempferol

Table S2. Effect of different concentrations of H_2O_2 on the viability of HepG2 cells.

	Concentration of H_2O_2 (μmol/mL)	Survival rate (%)
Control	0	100 ± 6.72^{A}
H_2O_2	100	109.48 ± 8.47^{A}
H_2O_2	200	109.08 ± 11.64^{A}
H_2O_2	400	102.95 ± 10.93^{A}
H_2O_2	800	59.99 ± 6.51^{B}
H_2O_2	1200	38.75 ± 7.52^{C}
H_2O_2	1600	22.52 ± 2.13^{D}
H_2O_2	3200	7.86 ± 9.09^{D}

Table S3. Effect of the incubation time of H_2O_2 on the viability of HepG2 cells.

	Time (h)	Survival rate (%)
H_2O_2 (800 μmol/mL)	1	113.18 ± 14.65^{A}
H_2O_2 (800 μmol/mL)	2	93.51 ± 13.06^{AB}
H_2O_2 (800 μmol/mL)	4	67.65 ± 7.47^{BC}
H_2O_2 (800 μmol/mL)	6	59.99 ± 6.51^{C}
H_2O_2 (800 μmol/mL)	8	58.12 ± 9.81^{C}

Table S4. Primer sequences for RT-qPCR

Primer name	Primer sequences (5'-3')
Gapdh-F	AACAGCAACTCCCACTCTTCC
Gapdh-R	TGGTCCAGGGTTTCTTACTCC
HO-1-F	CACATCCAAGCCGAGAATGC
HO-1-R	GTACAAGGAAGCCATCACCAG
GCLC-F	CACATCTACCACGCAGTCAAG
GCLC-R	CATCGCCTCCATTCAGTAACAA
GSTA2-F	CTTGATGCCAGCCTTCTGAC
GSTA2-R	TGCCAGGATGTAGGAACTTCTT
NQO1-F	ATGAAGGAGGCTGCTGTAGAG
NQO1-R	GCTAGAGATGACTCGGAAGGAT
Nrf2-F	CCTCAGCATGATGGACTTGGA
Nrf2-R	ACTTGTACCGCCTCGTCTG
